# Supplementary material for: Mutations in PCYT2 disrupt etherlipid biosynthesis and cause a complex hereditary spastic paraplegia
Source: Brain. 2019 Oct 22;142(11):3382–97. doi: 10.1093/brain/awz291 (PMC6821184; doi:10.1093/brain/awz291)
Supplement: awz291_Supplementary_Data [file awz291_supplementary_data.zip › awz291-Suppl_data/Supplementary_Data4.zip!/Supplementary information 4 - plasma lipidomics.zip!/Plasma lipidomics/Control vs Patient1/Statistical plots and data/TG species/Random forest.pdf]

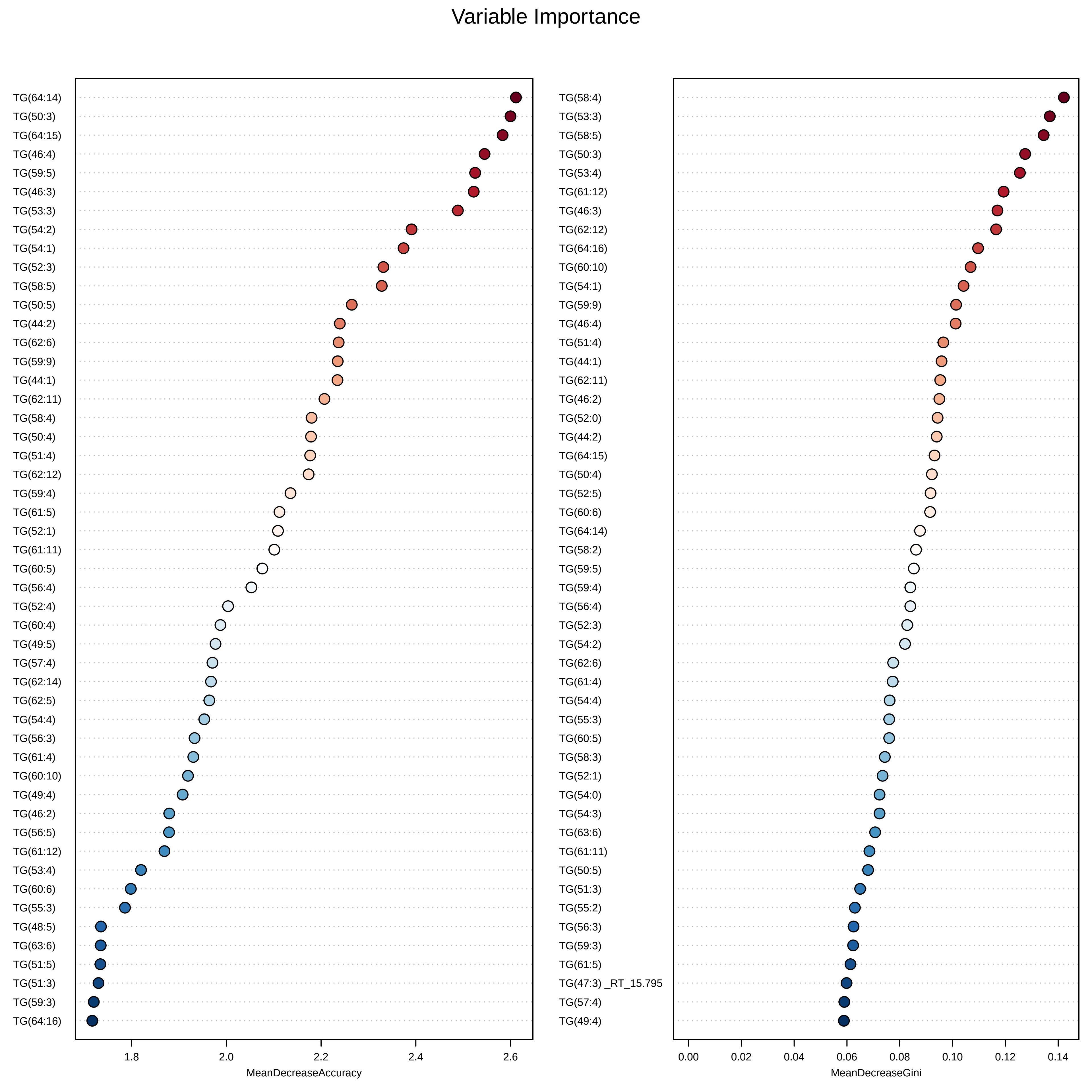

Variable Importance
TG(64:14)
TG(50:3)
TG(64:15)
TG(46:4)
TG(59:5)
TG(46:3)
TG(53:3)
TG(54:2)
TG(54:1)
TG(52:3)
TG(58:5)
TG(50:5)
TG(44:2)
TG(62:6)
TG(59:9)
TG(44:1)
TG(62:11)
TG(58:4)
TG(50:4)
TG(51:4)
TG(62:12)
TG(59:4)
TG(61:5)
TG(52:1)
TG(61:11)
TG(60:5)
TG(56:4)
TG(52:4)
TG(60:4)
TG(49:5)
TG(57:4)
TG(62:14)
TG(62:5)
TG(54:4)
TG(56:3)
TG(61:4)
TG(60:10)
TG(49:4)
TG(46:2)
TG(56:5)
TG(61:12)
TG(53:4)
TG(60:6)
TG(55:3)
TG(48:5)
TG(63:6)
TG(51:5)
TG(51:3)
TG(59:3)
TG(64:16)
1.8
2.0
2.2
2.4
2.6
MeanDecreaseAccuracy
TG(58:4)
TG(53:3)
TG(58:5)
TG(50:3)
TG(53:4)
TG(61:12)
TG(46:3)
TG(62:12)
TG(64:16)
TG(60:10)
TG(54:1)
TG(59:9)
TG(46:4)
TG(51:4)
TG(44:1)
TG(62:11)
TG(46:2)
TG(52:0)
TG(44:2)
TG(64:15)
TG(50:4)
TG(52:5)
TG(60:6)
TG(64:14)
TG(58:2)
TG(59:5)
TG(59:4)
TG(56:4)
TG(52:3)
TG(54:2)
TG(62:6)
TG(61:4)
TG(54:4)
TG(55:3)
TG(60:5)
TG(58:3)
TG(52:1)
TG(54:0)
TG(54:3)
TG(63:6)
TG(61:11)
TG(50:5)
TG(51:3)
TG(55:2)
TG(56:3)
TG(59:3)
TG(61:5)
TG(47:3) _RT_15.795
TG(57:4)
TG(49:4)
0.00
0.02
0.04
0.06
0.08
0.10
0.12
0.14
MeanDecreaseGini
